# Talar Body Fracture Treated by Hindfoot Endoscopic Reduction and Internal Fixation

**DOI:** 10.1155/2022/6183508

**Published:** 2022-05-16

**Authors:** Airi Shimmyo, Shota Morimoto, Toshiya Tachibana, Tomoya Iseki

**Affiliations:** Department of Orthopaedic Surgery, Hyogo College of Medicine, Hyogo, Japan

## Abstract

**Background:**

A talar body fracture is relatively rare. Surgical treatment for the fracture is generally indicated for the displaced fracture types and traditionally performed via open approaches. However, open approaches may not be able to achieve adequate exposure of the talus body owing to the difficulty in achieving reduction and fixation of the fracture. In this case report, we describe a case of talar body fracture treated by hindfoot endoscopic reduction and internal fixation. *Case report.* A 39-year-old man who was a carpenter complained of right hindfoot pain after he fell from a stepladder during work. Although plain radiographs of the right ankle showed no abnormal findings, noncontrast computed tomography demonstrated a fracture line from the lateral side of the posterior lateral process to the medial side of the talus body. In addition, the fracture line extended to the posterior ankle and subtalar joints, and the bone fragment was slightly displaced. We diagnosed him with the talar body fracture and performed operative treatments using hindfoot endoscopic reduction and internal fixation. At 14 weeks after the operation, he was able to return to work at the preinjury activity level without dysfunction of the ankle nor complications.

**Conclusion:**

We reported a case of talar body fracture treated by hindfoot endoscopic reduction and internal fixation. In this case, the hindfoot endoscopic technique provided visualization of the fracture site with less invasiveness and increased safety, which enabled proper reduction and internal fixation of the fracture site. Therefore, the patient was able to return to work at the preinjury activity level at 14 weeks after surgery without dysfunction of the ankle nor complications. This surgical technique may be a useful option for the talar body fracture.

## 1. Introduction

Talus fractures account for 3 to 5% of foot and ankle fractures, in particular, talar body fractures are relatively rare, accounting for only 7 to 38% of all talus fractures [1–6] This fracture commonly occurs in relatively active young adults and is often caused by high-energy axial loading injuries, such as car accidents, falls from a height, and other high-energy trauma [7] In general, conservative treatment is applied for completely nondisplaced fractures in CT scan, while surgical treatment involves anatomic reduction and internal fixation for displaced fractures [8, 9]. In surgical treatment, depending on the fracture pattern and location, the open approach methods such as an anteromedial, an anterolateral, a posteromedial, a posterolateral approach, or combinations of these approaches are commonly selected [8]. However, these approaches may not be able to achieve adequate exposure of the talus body owing to the difficulty in achieving reduction and fixation of the fracture [8] In addition, percutaneous screw fixation under fluoroscopy should be another option for less displaced fractures of the talar body [10] However, percutaneous technique has anatomic risks such as neurovascular bundle and FHL injuries due to a narrower safety zone for screw insertion [11, 12].

A 2-portal hindfoot endoscopic technique was first described by van Dijk et al. [13] in 2000. This technique has recently come to be used as a surgical approach for various pathologies located in the hindfoot, due to its advantages such as direct visualization of structures, less postoperative pain, a shorter functional recovery period, lower complication rates, and preservation of blood supply, and also because it is minimally invasive in comparison with traditional open approaches [14–17]. Additionally, the use of the hindfoot endoscopic technique allows visualization of the hindfoot structures and the screw insertion site, and so there is little risk of neurovascular bundle and FHL injuries as a result of screw insertion.

Here, we report a case of talar body fracture treated by hindfoot endoscopic reduction and internal fixation. The patient was able to return to work at the preinjury activity level at 14 weeks after surgery without complications, and no recurring symptoms nor refracture were present at the 2-year follow-up. Written informed consent was obtained from the patient for publishing this report.

## 2. Case Report

**Figure 1:**
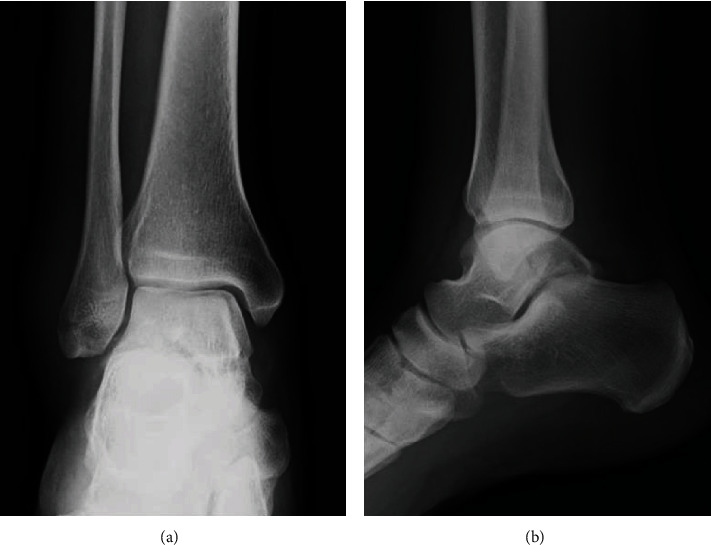
Plain radiographs of the right ankle showed no abnormal findings in (a) anteroposterior and (b) lateral views.

**Figure 2:**
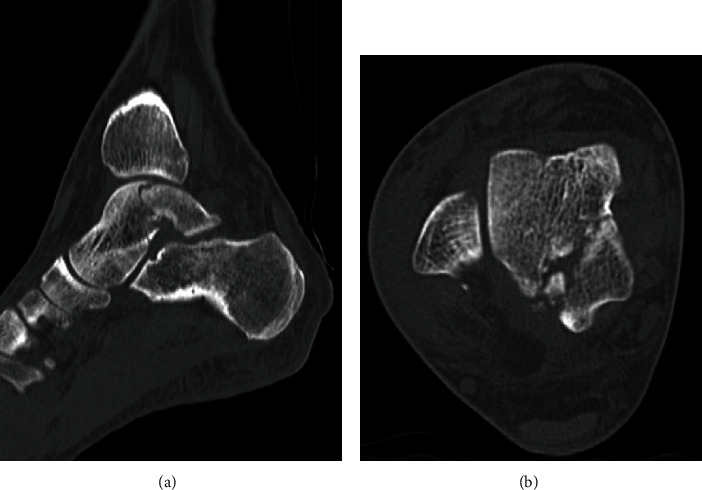
Preoperative noncontrast CT in (a) sagittal and (b) axial views demonstrated the fracture line from the lateral side of the posterior lateral process to the posteromedial side of the talus body. In addition, the fracture line extended to the posterior ankle and subtalar joints, and the bone fragment was slightly displaced.

A 39-year-old man who was a carpenter complained of right hindfoot pain after he fell from a stepladder during work. At the first visit to our hospital, he could not walk due to his right hindfoot pain. Physical examination revealed swelling, ecchymosis, and tenderness at the posterior aspect of his ankle. Plain radiographs of the right ankle showed no abnormal findings in anteroposterior and lateral views (Figure 1). Noncontrast computed tomography (CT) demonstrated a fracture line from the lateral side of the posterior lateral process to the medial side of the talus body. In addition, the fracture line extended to the posterior ankle and subtalar joints, and the bone fragment was slightly displaced (Figure 2). Based on the physical examination and radiological findings, the diagnosis of the talar body fracture was made. Because of the displaced bone fragments from the fracture, surgical treatment was applied for this case.

**Figure 3:**
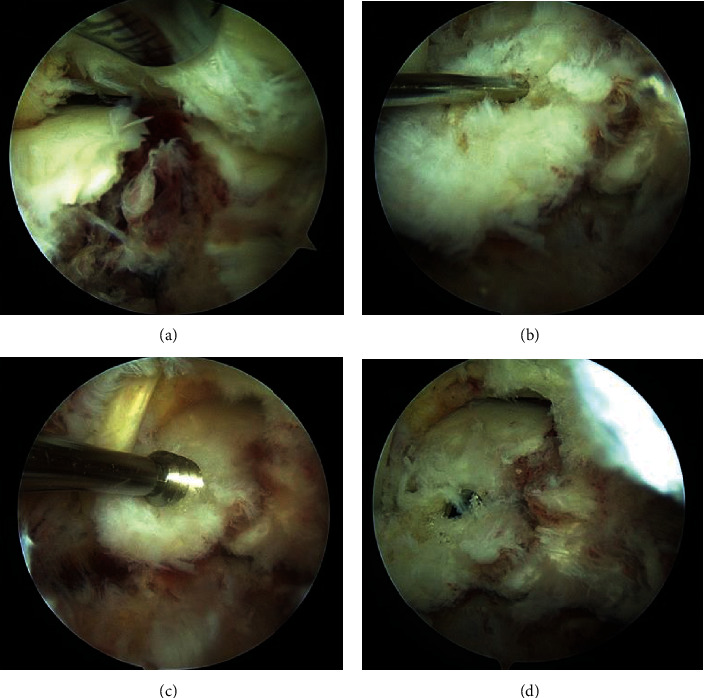
After the posterior aspect of the talus was observed using a 4.0-mm-diameter 30° arthroscope, and soft tissues around the talus including synovium, adipose tissue, and coagulation clot were removed with a 4.0-mm-diameter motorized shaver, the fracture site was confirmed (a). The fragment was reduced using a guidewire sleeve and fixed with two 0.8 mm guidewires from posteromedial portal (b). Through the guidewires (c), the fragment was fixed with two cannulated double-threaded screws (d).

**Figure 4:**
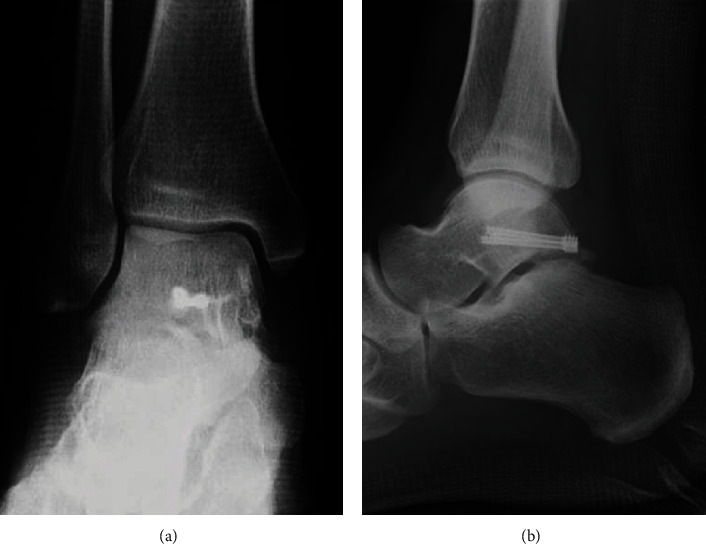
Postoperative plain radiograph in (a) anteroposterior and (b) lateral views showed that the fragment was reduced and fixed with two cannulated double-threaded screws.

The surgery was performed under general anesthesia in the prone position. A thigh air tourniquet and a fluoroscopy were used. Based on the hindfoot endoscopic technique reported by van Dijk et al. [13], posterolateral and posteromedial portals were created. The posterolateral portal was used as a viewing portal, and the posteromedial portal was used as a working portal for the motorized shaver and guide wires. First, the posterior aspect of the talus was observed using a 4.0-mm-diameter 30° arthroscope. Soft tissues around the talus including synovium, adipose tissue, and coagulation clot were removed with a 4.0-mm-diameter motorized shaver, and the fragment was easily reduced using a guidewire sleeve and fixed with two 0.8 mm guidewires. A lateral view of the ankle joint was visualized by a fluoroscopy in the prone position, and we confirmed the guidewires in an adequate position and the alignment after the reduction of the bone fragment. Through the guidewires, the fragment was fixed with two cannulated double-threaded screws (Double Thread Screw Japan Mini, Meira, Nagoya, Japan) (Figure 3). The wound was sutured, and the surgery was concluded (Figure 4).

**Figure 5:**
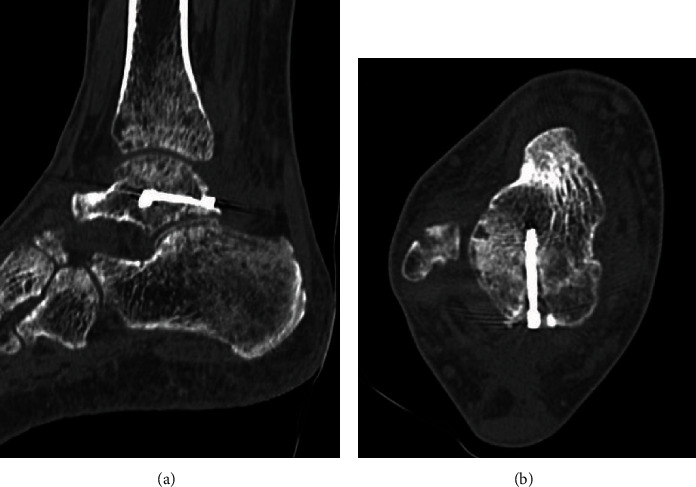
Noncontrast CT in (a) sagittal and (b) axial views at 14 weeks after the operation demonstrated complete bone union.

Postoperatively, a nonweightbearing short leg splint was applied for 2 weeks, and when the splint was removed, active and passive ranges of motion exercises of the ankle were initiated. Partial weightbearing was allowed at 6 weeks postoperatively, and full weightbearing at 10 weeks postoperatively. The patient was permitted to return to work after confirming consolidation of the fracture site on CT at 14 weeks after the operation (Figure 5). At 2 years after surgery, he was able to work as a carpenter without any complications nor symptoms, and the AOFAS score was 97.

## 3. Discussion

Talar body fractures are treated with either conservative or surgical treatment [9]. In general, conservative treatment is applied for completely nondisplaced fractures in CT scan, while surgical treatment involves anatomic reduction and internal fixation for displaced fractures [8, 9]. Additionally, percutaneous screw fixation should be another option for nondisplaced fractures in order to prevent redislocation and allows early rehabilitation such as range of motion exercises and weightbearing [9].

In surgical treatment for displaced fractures of the talar body, open approach methods, such as an anteromedial, an anterolateral, a posteromedial, a posterolateral approach, or a combination of these approaches are generally used depending on the fracture pattern and location [8]. However, these approaches might not be able to achieve adequate exposure of the talus body owing to the difficulty in achieving proper reduction and fixation of the fracture. Consequently, it would lead to ankle and subtalar arthritis. Previous reports have demonstrated that ankle and subtalar arthritis occurs at high rates after surgical treatment for talar body fractures. Spennacchio et al. [17] have reported 65% incidence of postoperative arthritis in the ankle joint and 34% incidence in the subtalar joint, and Sneppen et al. [18] showed that 95% of the cases had postoperative pain because of arthritic changes in the ankle and subtalar joints.

In addition, avascular necrosis (AVN) is a common complication in talar body fractures [9]. Ebraheim et al. [6] demonstrated 38% incidence of AVN after surgical treatment for talar body fractures and Lindvall et al. [19] operatively treated 26 cases of isolated, displaced talar neck and body fractures, reporting that the findings of AVN were seen in 13 cases (50%). Although the incidence of AVN in talar body fractures is reported to vary considerably, many authors agree that the degree of fracture displacement is associated with the incidence of AVN [20]. However, it has been reported that the incidence of AVN is related to not only the degree of displacement but also poor original blood supply in the talar body and the surgical approach [21]. Therefore, preservation of the remaining blood supply is important in surgical treatments for talar body fractures.

The hindfoot endoscopic technique is popular as a surgical approach for posterior ankle impingement syndrome (PAIS) and subtalar osteoarthritis (OA) [17]. Surgical treatments for these conditions have traditionally used an open approach [22, 23]. It has been reported that the hindfoot endoscopic approach for PAIS and subtalar OA has advantages such as lower complication rates, shorter functional recovery, less blood loss, less postoperative pain, and quicker union time compared to the traditional open approach [14–17]. Owing to the capability of the visualization of the hindfoot structures using a safe and minimally invasive technique, the hindfoot endoscopic approach has recently begun to be used for the treatment and diagnosis of other hindfoot pathologies including osteochondral lesions of the talus, talocalcaneal coalition, retrocalcaneal bursitis, synovial osteochondromatosis, pigmented villonodular synovitis, and talar fractures [17]. To our knowledge, there have only been three reports of talus body fractures treated by the hindfoot endoscopic technique [1, 24]. Ogut et al. [24] first described hindfoot endoscopic surgery for a case of a complex talus fracture involving the posterior part of the talar body and the posterior process. They treated the case with a combined excision of the posterolateral process and fixation of the fracture, and satisfactory results were obtained. Sitte et al. [1] treated 2 cases of a talar body shear fracture with osteosynthesis assisted by a hindfoot and subtalar arthroscopic technique. These reports suggest that the hindfoot endoscopic approach has the advantages of being able to visualize the fracture site and the insertion site of the screws by using a minimally invasive technique, which preserves the blood supply of the talar body and potentially decreases postoperative complications such as AVN [1, 24]. However, there were no cases that required anatomical reduction of the bone fragments in these reports, because they were nondisplaced and complex fractures.

In the present case, the bone fragment was relatively large, slightly displaced, and required anatomical reduction and internal fixation. Also, the fracture site extended to the posterior part of the talus body; therefore, the hindfoot endoscopic approach was selected. To our knowledge, this is the first report on a hindfoot endoscopic reduction and internal fixation for a talar body fracture. Because the hindfoot endoscopic approach was able to provide good visualization of the fracture site and the fracture pattern was a simple fracture type, proper reduction, internal fixation, and accurate screw positioning were attained, which led to good results without dysfunction or arthritis in the ankle and subtalar joints. Furthermore, a minimally invasive approach using the hindfoot endoscopic technique preserved blood supply in the talar body without complications such as AVN.

## 4. Conclusion

We reported a case of talar body fracture treated by hindfoot endoscopic reduction and internal fixation. In this case, the hindfoot endoscopic technique provided visualization of the fracture site with less invasiveness and increased safety, which enabled proper reduction and internal fixation of the fracture site. Therefore, the patient was able to return to work at the preinjury activity level at 14 weeks after surgery without dysfunction of the ankle nor complications such as arthritis and AVN. This surgical technique may be a useful option for the talar body fracture.

## Data Availability

The data used to support the findings of this study are included within the article.

## References

[1] Sitte W., Lampert C., Baumann P. (2012). Osteosynthesis of talar body shear fractures assisted by hindfoot and subtalar arthroscopy: technique tip. *Foot & Ankle International*.

[2] Buza J. A., Leucht P. (2018). Fractures of the talus: current concepts and new developments. *Foot and Ankle Surgery*.

[3] Fournier A., Barba N., Steiger V. (2012). Total talar fracture - Long-term results of internal fixation of talar fractures. A multicentric study of 114 cases. *Orthopaedics & Traumatology, Surgery & Research*.

[4] Von Knoch F., Reckord U., Von Knoch M., Sommer C. (2007). Fracture of the lateral process of the talus in snowboarders. *Journal of Bone and Joint Surgery. British Volume (London)*.

[5] Higgins T. F., Baumgaertner M. R. (1999). Diagnosis and treatment of fractures of the talus: a comprehensive review of the literature. *Foot & Ankle International*.

[6] Ebraheim N. A., Patil V., Owens C., Kandimalla Y. (2008). Clinical outcome of fractures of the talar body. *International Orthopaedics*.

[7] Sasaki M. H. (2014). Epidemiological study on talus fractures. *Revista Brasileira de Ortopedia*.

[8] Sundararajan S. R., Badurudeen A. A., Ramakanth R., Rajasekaran S. (2018). Management of talar body fractures. *Indian J Orthop.*.

[9] Rammelt S., Zwipp H. (2009). Talar neck and body fractures. *Injury*.

[10] Crate G., Robertson A., Martin A., Marlow N. J., Guryel E., Trompeter A. (2021). Talar neck and body fracture outcomes: a multicentre retrospective review. *European Journal of Orthopaedic Surgery and Traumatology*.

[11] Roberts L. E., Pinto M., Staggers J. R., Godoy-Santos A., Shah A., de Cesar Netto C. (2018). Soft tissue structures at risk with percutaneous posterior to anterior screw fixation of the talar neck. *Foot & Ankle International*.

[12] Beltran M. J., Mitchell P. M., Collinge C. A. (2016). Posterior to anteriorly directed screws for management of talar neck fractures. *Foot & Ankle International*.

[13] van Dijk C. N., Scholten P. E., Krips R. (2000). A 2-portal endoscopic approach for diagnosis and treatment of posterior ankle pathology. *Arthroscopy*.

[14] Georgiannos D., Bisbinas I. (2017). Endoscopic versus open excision of os trigonum for the treatment of posterior ankle impingement syndrome in an athletic population: a randomized controlled study with 5-year follow-up. *The American Journal of Sports Medicine*.

[15] Vilá Y., Rico J., Thies C. O., Sanchez G. P. (2016). Arthroscopic posterior subtalar arthrodesis: surgical technique. *Arthroscopy Techniques*.

[16] Yasui Y., Hannon C. P., Hurley E., Kennedy J. G. (2016). Posterior ankle impingement syndrome: a systematic fourstage approach. *World Journal of Orthopedics*.

[17] Spennacchio P., Cucchi D., Randelli P. S., van Dijk N. C. (2016). Evidence-based indications for hindfoot endoscopy. *Knee Surgery, Sports Traumatology, Arthroscopy*.

[18] Sneppen O., Christensen S. B., Krogsøe O., Lorentzen J. (1977). Fracture of the body of the talus. *Acta Orthopaedica Scandinavica*.

[19] Lindvall E., Haidukewych G., DiPasquale T., Herscovici D., Sanders R. (2004). Open reduction and stable fixation of isolated, displaced talar neck and body fractures. *The Journal of Bone and Joint Surgery. American Volume*.

[20] Saravi B., Lang G., Ruff R. (2021). Conservative and surgical treatment of talar fractures: a systematic review and meta-analysis on clinical outcomes and complications. *International Journal of Environmental Research and Public Health*.

[21] Prasarn M. L., Miller A. N., Dyke A. N., Helfet D. L., Lorich D. G. (2010). Arterial anatomy of the talus: a cadaver and gadolinium-enhanced MRI study. *Foot & Ankle International*.

[22] Coetzee J. C., Seybold J. D., Moser B. R., Stone R. M. (2015). Management of posterior impingement in the ankle in athletes and dancers. *Foot & Ankle International*.

[23] Easley M. E., Trnka H. J., Schon L. C., Myerson M. S. (2000). Isolated subtalar arthrodesis. *The Journal of Bone and Joint Surgery. American Volume*.

[24] Ogut T., Seyahi A., Aydingoz O., Bilsel N. (2009). A two-portal posterior endoscopic approach in the treatment of a complex talus fracture: a case report. *Journal of the American Podiatric Medical Association*.

